# Evaluation of patients with multiple sclerosis using reverse nutech functional score and expanded disability status scale after human embryonic stem cell therapy

**DOI:** 10.1186/s40169-016-0124-3

**Published:** 2016-10-20

**Authors:** Geeta Shroff

**Affiliations:** Nutech Mediworld, H-8, Green Park Extension, New Delhi, 110016 India

**Keywords:** Multiple sclerosis, Reverse nutech functional score (RNFS), Human embryonic stem cell (hESC), Expanded disability status scale (EDSS)

## Abstract

**Background:**

The expanded disability status scale (EDSS) is a validated and reliable tool to assess the extent of disabilities in patients with multiple sclerosis (MS). However, the use of this tool has been found to be limited in assessing various symptoms of MS that are important. Our study aimed at evaluating the efficacy of a new scoring system, reverse nutech functional score (RNFS) as compared to EDSS in assessing patients with MS treated with human embryonic stem cell (hESC) therapy.

**Methods:**

The MS patients were treated with hESC therapy for one treatment period. All the patients were evaluated with EDSS and RNFS at baseline and after the hESC therapy.

**Results:**

The study included a total of 24 MS patients with mean age of 45 year. The patients showed an improvement in parameters (sleeping disorders, paralysis, paraesthesia, myalgia, muscle weakness, memory, language, irritability, eye pain, depression and coordination, communication, breathing pattern, attention and appetite) associated with MS when evaluated with RNFS. This improvement went unnoticed when the patients were assessed with EDSS.

**Conclusion:**

RNFS can efficiently assess the effectiveness of hESC therapy in treating patients with MS. It could be a suitable scoring system for patients with MS as it can assess the slightest improvements in the patients. Use in other settings would be helpful in assessing its utility.

## Background

Multiple sclerosis (MS) is an autoimmune, chronic inflammatory demyelinating disease of the central nervous system (CNS) with genetic and environmental effects, among young and middle-aged adults [1, 2]. It affects around 2.5 million people worldwide and is the third most common neurologic disorder cited as the cause of disability [3]. According to the Centers for Disease Control and Prevention (CDC), approximately 85 % of the affected people have a relapsing-remitting course, characterized by an unpredictable course of exacerbations and remissions [4]. The diagnosis of MS is based on the lesions present in the CNS which appears in the different areas of brain [5].

The rate of growth of the disease is extremely mutable and uncertain, with an unclear etiology. Presently there is no cure, and only symptomatic therapy is available. Generally, oral or intravenous corticosteroids like methylprednisolone is used at a high dose in the routine therapy for acute attacks which results in a faster recovery from the disability within a duration of three to five days of course [6]. However, the corticosteroid therapy does not have a significant impact on the long term disability [7]. The improvement in therapy is measured by means of expanded disability status scale (EDSS) [8].

Basically, EDSS quantifies disability in eight functional systems (FS) and allows neurologists to assign a functional systems scores (FSS) in each of these systems. It consists of ordinal rating system ranging from 0 (normal neurological status) to 10 (death due to MS) in 0.5 increments interval (when reaching EDSS 1) [8]. EDSS also has a number of limitations. It is dependent on mobility of patient. It is subjective in certain areas (e.g., bowel and bladder function). It is also insensitive to small changes. Further, it does not present an accurate picture of the patient’s cognitive and functional abilities in performing activities of daily living (ADLs). It is nonlinear in terms of the time spent at various ranges of the scale [9].

We have previously published a case study of a patient with MS, who was treated with human embryonic stem cell therapy (hESC) and was assessed with EDSS before and after the therapy [10]. We have also developed a new functional, directional and positional scoring system, reverse nutech functional score (RNFS) to assess the patients with MS [11]. In the current study, we compared the two scoring systems, viz., RNFS and EDSS in patients with MS who underwent a single session of hESC therapy.

## Methods

The RNFS scoring system assesses a symptom based on five ordinal grades that runs in a direction of GOOD → BAD. The RNFS system is also useful in conducting probability based studies as these scores have been converted into numeric values. It is a numeric scale that scores all the known symptoms for patients with MS. RNFS for MS is a 36-point positional (i.e., symptoms were sub-graded with a specific score) and directional (i.e., from level 1 to 5) scoring system that can be used to assess or validate the diagnosis of patients with MS [11] whereas EDSS is a scoring system which quantifies disability in eight FS. An improvement in the symptoms was determined on the basis of two criteria referred as W1 and W2, where W1 = number of cases by symptom that score < best possible grade (BPG) at the time of admission or at baseline (BL) and reached BPG at the end of therapy (ET); W2 = number of cases by symptom that scored differently later by at least one grade of RNFS/EDSS as compared to the scores at BL. W1 represented the positive effect of hESC therapy to cure MS whereas W2 exhibited the effect, whether negative, neutral or positive, on the condition of a MS patient.

**Fig. 1:**
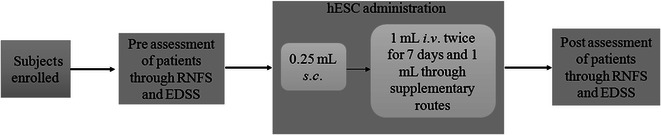
Schematic diagram for methodology. *RNFS* reverse nutech functional score; *EDSS* expanded disability status scale; *hESC* human embryonic stem cell; *sc* subcutaneous; *iv* intravenous

The establishment and characterization of hESCs were explained in our previous paper [12]. All the patients were assessed for hypersensitivity reactions to hESC by subcutaneous (s.c.) injection of 0.25 mL hESCs. Subsequent to safety evaluation, hESCs were administered via intramuscular (i.m.) route (twice daily) to “prime” the body, 1 mL hESCs (<16 million cells) were administered via intravenous (i.v.) route (twice every 7 days) to “home in” to the required area and 1–5 mL hESCs were administered via any of the supplemental routes including brachial plexus block, intrathecal, epidural catheter caudal, epidural and popliteal block and/or deep spinal muscle (every 7 days). All the patients received nasal sprays of 1 mL hESCs (3.5 million cells) twice a week to enhance the absorption of hESCs to the brain. The detailed methodology has been presented in Fig. 1. The safety studies of hESCs were explained in our previous paper [13].

In this study, the patients with MS were assessed with RNFS and EDSS. The RNFS/EDSS scores before and after the therapy were compared to assess which scoring system is better in evaluating the patients treated with hESC therapy. All the patients provided written informed consent prior to start of the study. The study was conducted in accordance with Declaration of Helsinki and approved by an independent Institutional Ethics Committee (IEC).

## Results

### Patients

A total of 24 patients with a mean age of 45 year were enrolled in the current study. The days of treatment in T1 varied from 42 to 84 days with a gap phase of 120–240 days.

### Patient wise status of EDSS grade at the ET

Out of 24 patients, only one patient reached the BPG at ET and 21 patients showed change in grade after the treatment (Table 1).

**Table 1:** Patient wise status of EDSS grade at the end of therapy

Patient code	Age (years)	Gender	Reached BPG	Change in grade
80806	29	M	No	Yes
80926	35	M	No	Yes
81128	64	M	No	No
80680	61	M	No	Yes
80710	43	M	No	Yes
80488	32	M	No	Yes
80499	61	M	No	Yes
80723	25	M	No	Yes
80981	56	F	Yes	Yes
80889	33	F	No	Yes
81238	30	F	No	Yes
81275	34	F	No	Yes
81114	54	F	No	Yes
81249	45	F	No	Yes
81296	30	F	No	Yes
80568	53	F	No	Yes
80626	54	F	No	Yes
80562	47	F	No	Yes
80768	57	F	No	Yes
81105	57	F	No	Yes
80673	32	F	No	Yes
80637	41	F	No	Yes
81187	53	F	No	No
81180	41	F	No	No

*EDSS* expanded disability status scale; *BPG* best possible grade

### Cases that scored less than BPG at BL and reached BPG afterwards

Number of affected parameters varied among the patients. Except five patients, all other patients showed an improvement (Table 2).

**Table 2:** Patient wise number of parameters that scored less than the best possible grade (<BPG) at baseline and reached BPG afterwards

Patient code	Number of affected parameters (<BPG)	Parameters improved at end of therapy; n (%)
80488	22	6 (27.3)
80499	12	6 (50.0)
80680	11	0
80710	15	9 (60.0)
80723	12	6 (50.0)
80806	2	1 (50.0)
80926	4	0
81128	10	3 (30.0)
80562	17	2 (11.8)
80568	17	1 (5.9)
80626	14	5 (35.7)
80637	15	2 (13.3)
80673	10	1 (10.0)
80768	13	1 (7.7)
80889	8	3 (37.5)
80981	5	3 (60.0)
81105	12	1 (8.3)
81114	6	0
81180	17	2 (11.8)
81187	7	0
81238	4	3 (75.0)
81249	9	1 (11.1)
81275	9	1 (11.1)
81296	8	0

*BPG* best possible grade

### Cases that scored differently later by at least one grade of RNFS

All the patients scored differently by at least one grade. One of the patient had 22 parameters that scored <BPG at BL and after the therapy, 20 (90.9 %) parameters improved by at least one grade Table 3.

**Table 3:** Patient wise number of cases who scored differently later by at least one grade of RNFS score at baseline

patient code	Number of affected parameters at BL (<BPG)	Alteration in score by at least one grade	
		No change; n (%)	Better; n (%)
80488	22	2 (9.1)	20 (90.9)
80499	12	3 (25.0)	9 (75.0)
80680	11	0	11 (100.0)
80710	15	3 (20.0)	12(80.0)
80723	12	3 (25.0)	9 (75.0)
80806	2	1 (50.0)	1 (50.0)
80926	4	2 (50.0)	2 (50.0)
81128	10	4 (40.0)	6 (60.0)
80562	17	0	17 (100.0)
80568	17	4 (23.5)	13 (76.5)
80626	14	4 (28.6)	10 (71.4)
80637	15	9 (60.0)	6 (40.0)
80673	10	5 (50.0)	5 (50.0)
80768	13	7 (53.8)	6 (46.2)
80889	8	3 (37.5)	5 (62.5)
80981	5	0	5 (100.0)
81105	12	6 (50.0)	6 (50.0)
81114	6	5 (83.3)	1 (16.7)
81180	17	10 (58.8)	7 (41.2)
81187	7	4 (57.1)	3 (42.9)
81238	4	0	4 (100.0)
81249	9	6 (66.7)	3 (33.3)
81275	9	8 (88.9)	1 (11.1)
81296	8	6 (75.0)	2 (25.0)

*RNFS* reverse nutech functional score; *BL* baseline; *BPG* best possible grade

### Parameters that scored less than BPG at BL and reached BPG afterwards

The scores for cases have also been categorized based on the affected parameters. Muscle weakness, an important symptom of MS, was scored to be as <BPG in all the cases at BL. At ET, 3 (12.5 %) patients reached BPG. Fatigue was scored as <BPG in 22 cases at BL, 8 (36.4 %) of them reached BPG at ET. The scores of all other parameters are presented in Table 4.

**Table 4:** Parameter wise number of patients who scored less than the best possible grade (<BPG) at baseline and reached BPG afterwards

Parameters	Affected patients (<BPG)	Improved patients at end of therapy; n (%)
Appetite	7	7 (100.0)
Attention	3	3 (100.0)
Balance—eyes closed in straight line	26	1 (3.8)
Balance—eyes open in straight line	24	3 (12.5)
Bladder—control	12	2 (16.7)
Bladder—sensation	8	2 (25.0)
Bowel—control	6	1 (16.7)
Bowel—sensation	4	2 (50.0)
Breathing pattern—Brady	1	1 (100.0)
Communication—speech	4	2 (50.0)
Coordination	14	0
Deformity	8	1 (12.5)
Depression	6	4 (66.7)
Double-vision + color blindness	5	1 (20.0)
Ears—hearing	3	3 (100.0)
Eye pain	2	2 (100.0)
Fatigue	22	8 (36.4)
Floaters	2	1 (50.0)
Irritability	7	4 (57.1)
Language	3	1 (33.3)
Limb tremors	15	6 (40.0)
Memory	7	5 (71.4)
Muscle weakness	27	3 (11.1)
Myalgia	11	5 (45.5)
Orientation—yes or no	1	1 (100.0)
Pain	18	7 (38.9)
Paralysis	5	0
Paraesthesia	11	5 (45.5)
Physical—drooling	1	1 (100.0)
Sitting	18	5 (27.8)
Sleep disorder—hypersomnia	4	4 (100.0)
Sleep disorder—hyposomnia	3	2 (66.7)
Stiffness	19	10 (52.6)
Tingling	7	3 (42.9)
Tinnitus	3	3 (100.0)
Vision—blurring	11	3 (27.3)

*BPG* best possible grade

## Discussion

In this study, an improvement in condition of the patients was evaluated more efficiently with RNFS system by highlighting even the slightest improvement. The levels in RNFS run in direction 1 (Good) → 5 (Bad) and patients could be placed in these levels on the basis of their symptoms and functional limitations.

Gray and Butzkueven stated that EDSS is subjective in certain areas (e.g., bowel and bladder function). It is also insensitive to small changes. Further, it does not present an accurate picture of the patient’s cognitive abilities and functional abilities in performing ADLs. It is nonlinear in terms of the time spent at various ranges of the scale [14]. Moreover, the practitioners and staff of our institute observed that EDSS emphasize mainly on the mobility of MS patients. The system is unable to assess other important parameters such as; sleeping disorders, paralysis, paraesthesia, myalgia, muscle weakness, memory, language, irritability, eye pain, depression, coordination, communication, breathing pattern, attention and appetite that were important to gauge in a patient with MS.

**Fig. 2:**
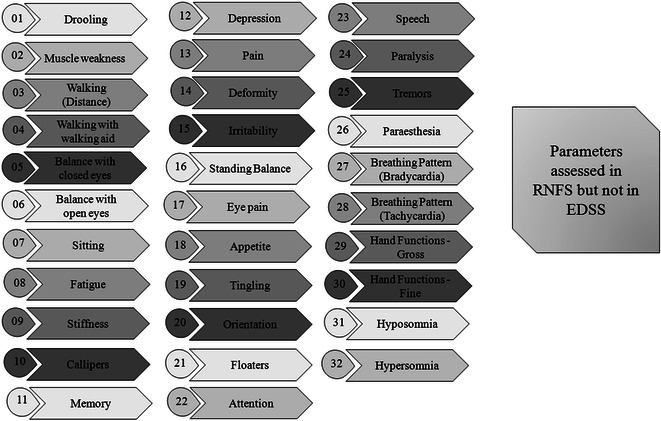
Parameters covered by reverse nutech functional score but not by expanded disability status scale. *RNFS* reverse nutech functional score; *EDSS* expanded disability status scale

Schwid et al. reported in the study for comparing limitations of walking ability in MS patients, the EDSS and ambulation index (AI) were less sensitive to change than the D max (the maximum distance that a person can go) and T8 (time to walk 8 m) [15]. Similarly, Vaney et al. reported lesser changes in EDSS than in the Rivermead mobility index (RMI), the AI and the 10 m walking time test [16]. Hohol et al. reported a lower sensitivity to change in EDSS compared with disease steps [17]. Healy et al. conducted a study to assess the sustained disease progression in relapsing-remitting multiple sclerosis (RRMS) with EDSS. The author found, between 15.8 and 42.2 % of patients experienced sustained progression based on the definitions using EDSS as the outcome, but nearly 50 % of these patients failed to maintain sustained progression for the duration of follow-up. The author concluded that short-term changes in the EDSS scores may not be an accurate marker of irreversible change in RRMS [18]. It has been difficult for clinicians to make normative evaluations of patient’s motor potency with EDSS. However, RNFS system is a functional and parametric system that assesses a patient with MS on the basis of all the parameters that were associated with MS. In our study, RNFS identified even the smallest change in the parameters of MS. Figure 2 show the list of disease related parameters that are assessed by RNFS but not by EDSS.

Use of hESC therapy has generally led to a concern because of clinical non-viability in culturing of cells in a xeno-free environment. However, our institution uses a patented methodology [19] for the extraction, isolation and maintenance of hESCs [12, 13]. The ability of stem cells to proliferate and to reconstruct the damaged parts offers excellent possibilities [20]. Previous studies have shown the potential of stem cells to migrate at the injury site and commence host repair and healing via the direct or indirect cell-signalling. In the course of brain injury, stem cells initiate neuroprotection and neural repair by inflammatory suppression, causing tissue reconstruction and avert cell damage. We have also reported the efficacy of hESCs in patients with MS [10]. The current study highlights the effectiveness of hESC therapy (as per the findings of RNFS and EDSS assessment) in the treatment of patients with MS. However, the assessment of MS lesions using gadolinium dye is warranted. Advances in this field are required to substantiate the efficacy results.

## Conclusion

The RNFS is a solitary classification system for the patients of all ages with diminished complexity of the assessment system for the practitioners. So, we conclude that the newly developed RNFS is a unique tool can be used to gauge the betterment of patients receiving hESC therapy. Thus, this newly developed classification, functional and evaluation system can be used globally to help the patients with MS.

## Abbreviations

MS: multiple sclerosis
CNS: central nervous system
CDC: Centers of Disease Control and Prevention
EDSS: expanded disability status scale
FS: functional systems
FSS: functional systems scores
ADL: activities of daily living
hESC: human embryonic stem cell
RNFS: reverse nutech functional score
BPG: best possible grade
BL: baseline
ET: end of therapy
IEC: Institutional Ethics Committee
AI: ambulation index
RMI: Rivermead mobility index
RRMS: relapsing-remitting multiple sclerosis
