# Expression of Concern to: Evaluation of patients with multiple sclerosis using reverse nutech functional score and expanded disability status scale after human embryonic stem cell therapy

**DOI:** 10.1186/s40169-017-0167-0

**Published:** 2017-09-18

**Authors:** Geeta Shroff

**Affiliations:** Nutech Mediworld, H‑8, Green Park Extension, New Delhi, 110016 India

## Expression of Concern: Clin Trans Med (2016) 5:43 DOI 10.1186/s40169-016-0124-3

The Editors-in-Chief of *Clinical and Translational Medicine* are issuing an editorial expression of concern to alert readers that concerns have been raised regarding the ethics of this study [1]. Appropriate editorial action will be taken once this has been fully investigated. The author disagrees with this notice.
